# Supplementary material for: Glycyl-tRNA Synthetase (GARS) Expression Is Associated with Prostate Cancer Progression and Its Inhibition Decreases Migration, and Invasion In Vitro
Source: Int J Mol Sci. 2023 Feb 21;24(5):4260. doi: 10.3390/ijms24054260 (PMC10001614; doi:10.3390/ijms24054260)
Supplement: Supplementary file 1 [file ijms-24-04260-s001.zip › ijms-2151520-supplementary.pdf]

International Journal of
Molecular Sciences

MDPI

Supplementary material

Glycyl-tRNA Synthetase (GARS) Expression Is Associated with Prostate Cancer Progression and Its Inhibition Decreases Migration, and Invasion In Vitro

Ealia Khosh Kish [1,2], Yaser Gamallat [1,2], Muhammad Choudhry [1,2], Sunita Ghosh [3,4], Sima Seyedi [2] and Tarek A. Bismar [1,2,5,6,*]

1 Department of Pathology and Laboratory Medicine, Cumming School of Medicine, University of Calgary, Calgary, AB T2N 4N1, Canada
2 Departments of Oncology, Biochemistry and Molecular Biology, Cumming School of Medicine, Calgary, AB T2N 4N1, Canada
3 Department of Medical Oncology, Faculty of Medicine and Dentistry, University of Alberta, Edmonton, AB T6G 2R7, Canada
4 Departments of Mathematical and Statistical Sciences, University of Alberta, Edmonton, AB T6G 2J5, Canada
5 Tom Baker Cancer Center and Arnie Charbonneau Cancer Institute, Calgary, AB T2N 4N1, Canada
6 Prostate Cancer Center, Rockyview General Hospital, Calgary, AB T2V 1P9, Canada
* Correspondence: tabismar@ucalgary.ca; Tel.: +1-403-943-8430; Fax: +1-403-943-3333

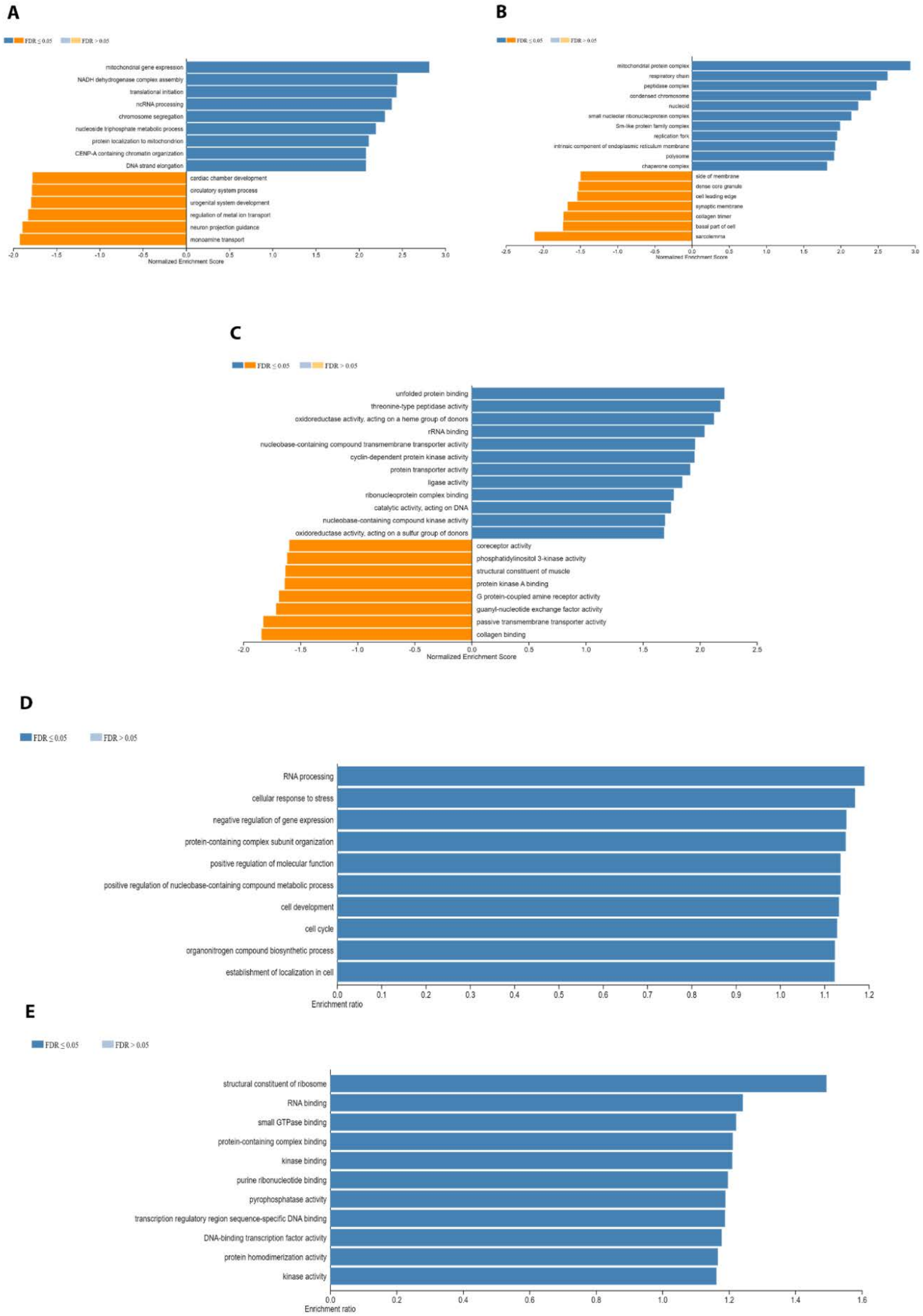

Figure S1. ***GARS*** **Overrepresentation Enrichment Analysis and Gene Set Enrichment Analysis.** Bar plots showing positively and negatively correlated genes using Overrepresentation Enrichment Analysis (ORA) for **(A)** Biological Processes, **(B)** Cellular Components and **(C)** Molecular Functions. Data was analyzed using Pearson Correlation and top 25 were chosen based on FDR rank (FDR ≤

0.05). **(D,E)** Bar plots showing correlated genes using Gene Set Enrichment Analysis (GSEA) for **(D)** Biological Processes and **(E)** Molecular Functions. Data was analyzed using Pearson Correlation, minimum 10 genes, 1000 stimulations and ranked based on FDR.

Table S1. List of Antibodies.

No	Antibody	Catalog number	Supplier	Western Blot Dilution
1	**Anti-GARS antibody (WB)**	**15831-1-AP**	**Protein tech**	**1:1000**
2	**Mouse anti-GAPDH antibody**	**60004-1-Ig**	**Protein tech**	**1:1000**
3	**Anti-Cyclin B1 antibody**	**Y106**	**Abcam**	**1:1000**
4	**Anti-P-PDK antibody**	**S241**	**Cell Signaling**	**1:1000**
5	**Mouse anti-Rabbit IgG antibody**	**7076S**	**Cell Signaling**	**1:1000**
6	**Rabbit anti-Mouse IgG antibody**	**7074S**	**Cell Signaling**	**1:1000**
7	**Anti-GARS antibody (IHC)**	**HPA019097**	**Sigma-Aldrich**	**1:500**
